# Non‐Invasive Detection of Early‐Stage Fatty Liver Disease via an On‐Skin Impedance Sensor and Attention‐Based Deep Learning

**DOI:** 10.1002/advs.202400596

**Published:** 2024-06-17

**Authors:** Kaidong Wang, Samuel Margolis, Jae Min Cho, Shaolei Wang, Brian Arianpour, Alejandro Jabalera, Junyi Yin, Wen Hong, Yaran Zhang, Peng Zhao, Enbo Zhu, Srinivasa Reddy, Tzung K. Hsiai

**Affiliations:** ^1^ Department of Medicine David Geffen School of Medicine University of California Los Angeles Los Angeles CA 90095 USA; ^2^ Department of Bioengineering, Henry Samueli School of Engineering and Applied Sciences University of California Los Angeles Los Angeles CA 90095 USA; ^3^ Department of Medicine Greater Los Angeles Veterans Affairs (VA) Healthcare System Los Angeles CA 90073 USA; ^4^ Department of Materials Science and Engineering University of California Los Angeles Los Angeles CA 90095 USA; ^5^ Department of Molecular and Medical Pharmacology University of California Los Angeles Los Angeles CA 90095 USA

**Keywords:** attention mechanism, deep learning, electrical impedance, nonalcoholic fatty liver disease, on‐skin sensor

## Abstract

Early‐stage nonalcoholic fatty liver disease (NAFLD) is a silent condition, with most cases going undiagnosed, potentially progressing to liver cirrhosis and cancer. A non‐invasive and cost‐effective detection method for early‐stage NAFLD detection is a public health priority but challenging. In this study, an adhesive, soft on‐skin sensor with low electrode‐skin contact impedance for early‐stage NAFLD detection is fabricated. A method is developed to synthesize platinum nanoparticles and reduced graphene quantum dots onto the on‐skin sensor to reduce electrode‐skin contact impedance by increasing double‐layer capacitance, thereby enhancing detection accuracy. Furthermore, an attention‐based deep learning algorithm is introduced to differentiate impedance signals associated with early‐stage NAFLD in high‐fat‐diet‐fed low‐density lipoprotein receptor knockout (*Ldlr^−/−^*) mice compared to healthy controls. The integration of an adhesive, soft on‐skin sensor with low electrode‐skin contact impedance and the attention‐based deep learning algorithm significantly enhances the detection accuracy for early‐stage NAFLD, achieving a rate above 97.5% with an area under the receiver operating characteristic curve (AUC) of 1.0. The findings present a non‐invasive approach for early‐stage NAFLD detection and display a strategy for improved early detection through on‐skin electronics and deep learning.

## Graphical abstract

The present work develops an adhesive on‐skin impedance sensor with a deep learning algorithm for detecting early‐stage nonalcoholic fatty liver disease (NAFLD), achieving an accurate rate above 97.5% with an area under the receiver operating characteristic curve of 1.0. This research represents a significant step forward in non‐invasive NAFLD detection, leveraging the latest advancements in on‐skin electronics and deep learning.

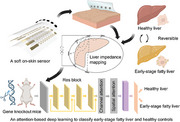

## 1 Introduction

Non‐alcoholic fatty liver disease (NAFLD) is the most common liver disease worldwide and one of the leading causes of acute coronary syndrome and stroke.^[^ ^1^ ^]^ A high‐fat diet increases fatty infiltrate in the liver, and hepatocytes (liver cells) have a limited capacity to metabolize excess fat, leading to lipid accumulation and NAFLD.^[^ ^2^ ^]^ The global prevalence of NAFLD is ≈ 25.24%, with the highest rates found in industrialized nations such as the United States.^[^ ^3^ ^]^ Most patients with fatty liver are undiagnosed, missing the therapeutic window to prevent cardiovascular disease, liver cirrhosis, and cancer.^[^ ^4^ ^]^ Thus, there is an unmet clinical need to detect the early stage of NAFLD.

While a liver biopsy remains the gold standard for diagnosing NAFLD, it is invasive and prone to bleeding risk.^[^ ^2a^ ^]^ Alternative imaging modalities, such as ultrasound, often fail to detect fatty liver at an early stage due to their relatively low resolution and sensitivity. An individual remains asymptomatic at the early and reversible fatty liver stage.^[^ ^4^, ^5^ ^]^ Notably, the early stages of NAFLD present a window of reversibility through targeted dietary and lifestyle interventions. In this context, there is an urgent need to develop non‐invasive and cost‐effective sensors for early detection and intervention.

The measured impedance consists of electrode‐skin contact impedance and body impedance.^[^ ^6^ ^]^ We aimed to explore on‐skin impedance sensors that could detect the changes in electrical impedance of fatty infiltration in the liver.^[^ ^7^ ^]^ At low frequencies, the lipid bilayers impede the current flow, resulting in high impedance. At high frequencies, the bilayers serve as imperfect capacitors, resulting in tissue and fluid‐dependent impedance. Fat‐free tissue, such as skeletal muscle, carries a high‐water content, ions, and protein content, allowing for efficient electrical conductivity.^[^ ^8^ ^]^ In contrast, fat‐infiltrated tissue, such as fatty liver, is anhydrous with reduced conductivity. These properties provide the basis for applying the multi‐frequency impedance technique to measure fatty infiltration of the liver.^[^ ^6^, ^9^ ^]^

However, high electrode‐skin contact impedance remains a barrier to acquiring accurate impedance signals for detecting fatty infiltration in the liver.^[^ ^6^, ^7^, ^10^ ^]^ Nanomaterials such as graphene quantum dots (GQDs), with their sp^2^‐bonded carbon structures and negatively charged oxygen‐rich functional groups, have demonstrated the feasibility of reducing electrode‐skin contact impedance.^[^ ^11^ ^]^ Electrochemical reduction techniques allow for precise modulation of the sp^2^ domains and negatively charged functional groups of GQDs.^[^ ^12^ ^]^ This fine‐tuning enables the rGQDs to effectively immobilize platinum nanoparticles (PtNPs) on sensing electrodes, thereby significantly increasing the capacitive double‐layer (C_dl_) to reduce electrode‐skin contact impedance.^[^ ^13^ ^]^

Deep learning algorithms play a crucial role in identifying the intricate patterns within datasets by employing the backpropagation technique to adjust parameters across layers iteratively.^[^ ^14^ ^]^ The attention‐based mechanism in deep learning algorithms can emulate the human cognitive process by pinpointing the regions of interest within the data.^[^ ^15^ ^]^ Integrating the attention mechanism into the deep learning algorithm is anticipated to enable more accurate detection of early‐stage NAFLD by focusing on the vital features within the dataset.^[^ ^16^ ^]^

This study proposed a non‐invasive and cost‐effective approach for detecting early‐stage NAFLD in high‐fat diet‐fed low‐density lipoprotein receptor knockout (*Ldlr^−/−^*) mice. To fabricate an adhesive, soft, and on‐skin sensor with a low electrode‐skin contact impedance, we seamlessly integrated the soft poly(styrene‐b‐isoprene‐b‐styrene) (SIS) block copolymer, serpentine conductive gold connections, medical‐grade adhesive silicone gels (Silbione RT Gel 4717 A/B), and platinum nanoparticles‐reduced graphene quantum dots (PtNPs@rGQDs) coating. Furthermore, we introduced an attention mechanism into the deep learning algorithm to extract relevant features associated with early‐stage NAFLD in the impedance dataset obtained from the fabricated sensor. We demonstrate a detection accuracy for early‐stage NAFLD above 97.5%, with an area under the receiver operating characteristic curve (AUC) of 1.0.

## 2 Results

### 2.1 A Strategy to Detect Early‐Stage NAFLD via an On‐Skin Impedance Sensor

**Figure 1:**
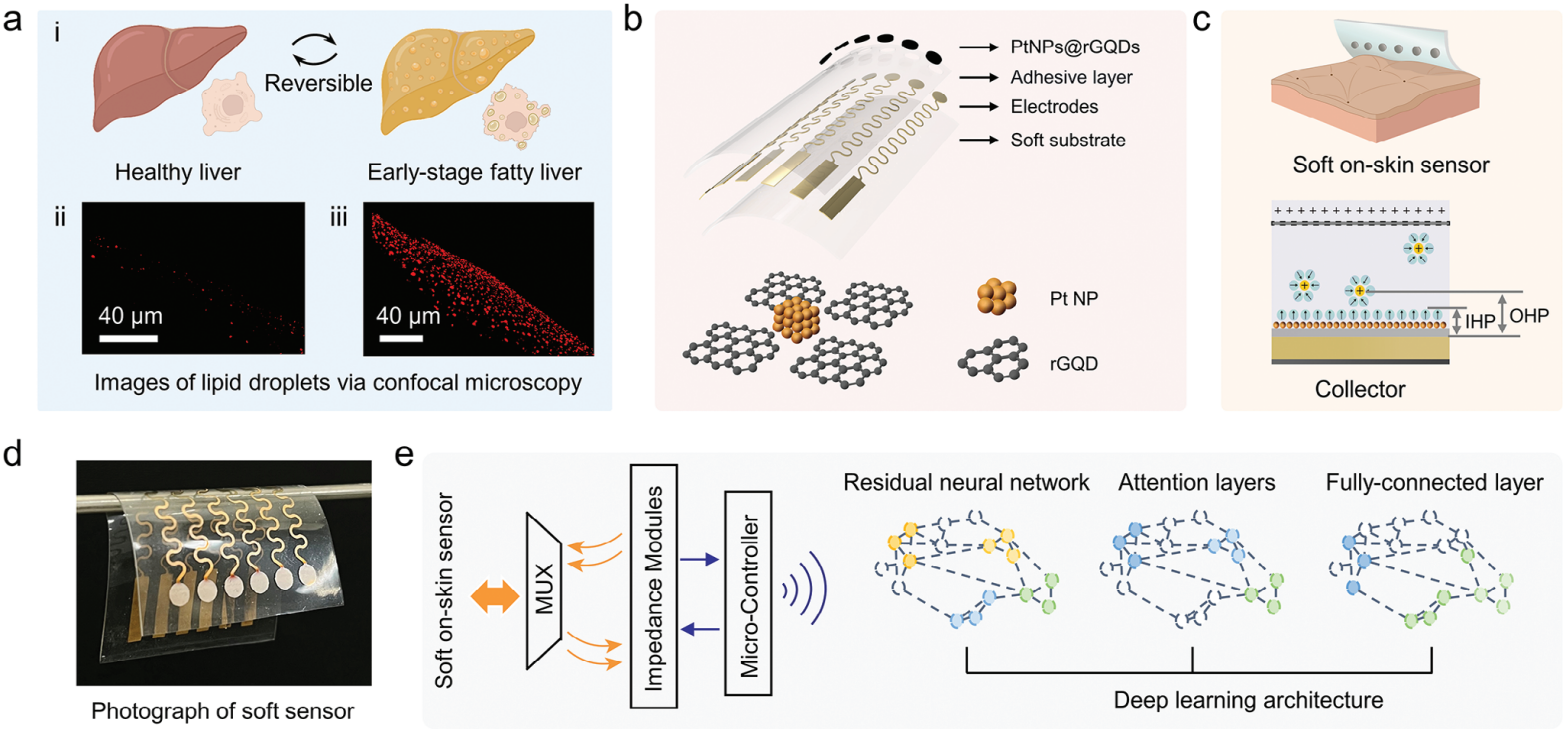
Non‐invasive detection approach for early‐stage NAFLD. a) Comparative confocal microscopy images illustrate lipid droplet accumulation in a healthy liver versus early‐stage NAFLD. b) Schematic representation of the on‐skin sensor, featuring a soft SIS substrate, patterned serpentine gold connections, an adhesive encapsulation layer, and PtNPs@rGQDs sensing electrodes. c) Detailed view of the capacitance increase facilitated by PtNPs@rGQDs sensing electrodes, including the Inner Helmholtz Plane (IHP) and Outer Helmholtz Plane (OHP). d) A photograph of the soft on‐skin sensor. e) An attention‐based deep learning model designed to extract classification features from impedance data obtained from the multiplexed on‐skin sensor.

Herein, we propose a non‐invasive approach for detecting early‐stage NAFLD employing a novel on‐skin impedance sensor. The fabricated impedance sensor, designed to measure alternating current (AC) electrical properties of liver tissues, offered a safer alternative to traditional invasive biopsy methods. To build an early‐stage NAFLD dataset, we exposed *Ldlr^−/−^* mice to a high‐fat diet regimen for four weeks.^[^ ^17^ ^]^ By utilizing the *Ldlr^−/−^* mice, which are genetically predisposed to high cholesterol, we manipulated the progression of NAFLD by administering a high‐fat diet regimen over time. After the non‐invasive measurements using the fabricated impedance sensor, we employed histological analysis involving Hematoxylin and Eosin (H&E) and Oil Red O staining along with image processing (Videos S1 and S2, Supporting Information) to quantify the distribution of lipid droplets in liver tissues.^[^ ^18^ ^]^ The staining and image analysis techniques ensure high accuracy in quantifying lipid accumulation in liver tissues and identifying early‐stage NAFLD. Utilizing 3D confocal microscopy, we observed significant visual contrasts between the hepatic profiles of *Ldlr^−/−^* mice affected by diet‐induced alterations and healthy controls, as depicted in **Figure** **1**a, Videos S1 and S2 (Supporting Information). Notably, the *Ldlr^−/−^* group exhibited a substantial accumulation of lipid droplets following the dietary intervention.

To facilitate the detection of early‐stage NAFLD using an on‐skin impedance sensor, we utilized a multi‐frequency electrical impedance technique to record both resistive and reactive components of the bioelectrical signal traversing the liver.^[^ ^19^ ^]^ The measurement accuracy of liver impedance can be adversely affected by electrode‐skin contact impedance between the sensor and skin (Figure S1, Supporting Information). To reduce electrode‐skin contact impedance, we engineered an adhesive and soft impedance sensor with highly conductive and capacitive sensing electrodes (Figure 1b). The soft SIS substrate facilitated optimal contact between the sensor and the skin.^[^ ^20^ ^]^ In contrast, the adhesive silicone gels serve the dual functions of insulating the conductive pathways and ensuring sustained adhesion on the skin (Figure 1d). The functionalization of electrode surfaces by integrating rGQDs with PtNPs leads to a notable increase in capacitance at the inner Helmholtz plane (IHP), effectively reducing electrode‐skin contact impedance between the sensor and skin (Figure 1c).^[^ ^19^, ^21^ ^]^

To fulfill the binary classification of early‐stage NAFLD, we employed a deep learning architecture that includes residual neural network (ResNet), attention layers, and fully‐connected layer to extract relevant features associated with early‐stage NAFLD in the impedance dataset obtained from the fabricated sensor (Figure 1e).^[^ ^22^ ^]^

### 2.2 A Soft, Adhesive, and On‐Skin Impedance Sensor

**Figure 2:**
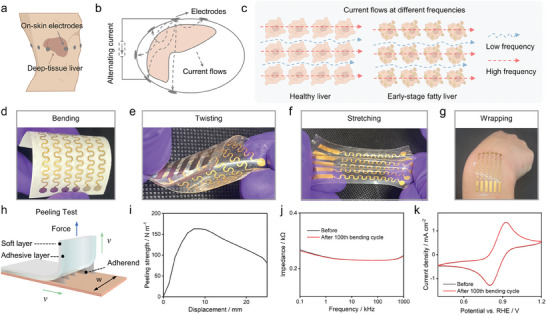
Fabrication and functional testing of a soft impedance sensor. a) Localization of electrodes on the human body. b) Diagram depicting the electrical current pathways. c) Graphical representation of current flow patterns at different frequencies, contrasting between a healthy liver and an early‐stage NAFLD. d–g) Sequential illustrations of the soft sensor subjected to mechanical deformations: d) bending, e) twisting, f) stretching, and g) conforming to surface contours. h) Illustration of a peeling test. i) Force‐displacement curve illustrating the adhesive sensor detachment from the skin. j,k) Comparative analysis of electrical impedance and cyclic voltammetry before and after the 100th bending test cycle, assessing durability and functional integrity.

We employed a multi‐frequency electrical impedance technique to record impedance signals associated with the liver using an on‐skin sensor.^[^ ^6^, ^23^ ^]^ We fabricated a soft sensor on the skin near the liver (**Figure** **2**a,b). Biological tissues exhibit capacitive properties, manifesting frequency‐dependent electrical impedance in the presence of alternating currents. Fat‐free tissues are more conductive due to their high‐water content, in contrast to the reduced conductivity of anhydrous fatty tissues. These differential properties were exploited to differentiate between a healthy liver and early‐stage NAFLD via an on‐skin impedance sensor (Figure 2c).

To facilitate the non‐invasive detection of early‐stage NAFLD, we engineered an on‐skin sensor deformable to irregular objects, incorporating a four‐layer design to optimize softness, mechanical resilience, adhesiveness, and electrical impedance characteristics. The impedance sensor exhibited high softness, as illustrated in Figure 2d–g, including bending (d), twisting (e), stretching (f), and wrapping (g), facilitating favorable contact with irregular objects.

The substrate layer, built from an SIS block copolymer, is selected for its superior film‐forming ability, elasticity, and biocompatibility.^[^ ^24^ ^]^ The subsequent layer is composed of gold wires, sputtered into a serpentine configuration to improve long‐term mechanical integrity. The adhesive silicone gel was sprayed onto the gold wires in the serpentine configuration to insulate them and enhance dermal contact. To quantitatively evaluate the adhesive properties of the impedance sensor, a standard peeling test adhering to the prescribed rate of 50 mm/min was employed.^[^ ^25^ ^]^ The detailed schematic of this testing procedure is delineated in Figure 2h, and corresponding bonding strength measurements are presented in Figure 2i. The peeling strength was calculated by the ratio of the peeling force (F) to the film width (w). The peak peeling strength reached 163 N/m, demonstrating that the fabricated impedance sensor had high adhesiveness to ensure stable contact between the sensor and skin for impedance recordings. The structural robustness of the sensor was evaluated through extensive bending tests. These tests revealed that the variances in electrical impedance signals (Figure 2j) and cyclic voltammetry (Figure 2k) remained under 1% even after the 100th bending test cycle, demonstrating excellent mechanical stability.

### 2.3 Synthesis of PtNPs@rGQDs to Reduce Electrode‐Skin Contact Impedance

**Figure 3:**
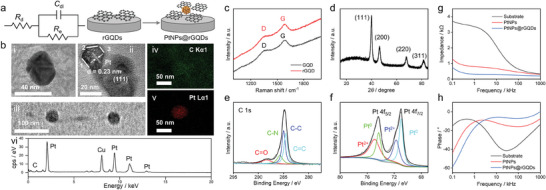
Structural characterizations of PtNPs@rGQDs. (a) Randles equivalent circuit and schematic representation of the two‐step synthesis process for PtNPs@rGQDs sensing electrodes. b) High‐resolution TEM images of PtNPs@rGQDs (i, ii, and iii), elemental mapping images for carbon (C, iv) and platinum (Pt, v), and energy spectrum to confirm the existence of C and Pt. The TEM grid was made from copper, and a Cu element peak was observed. c) Raman spectroscopy analysis of GQDs and rGQDs. d) XRD pattern of PtNPs@rGQDs. e,f) XPS spectra of the C 1s (e) and Pt 4f (f) regions. g,h) Comparative plots of electrical impedance g) and phase angle h) measurements for PtNPs and PtNPs@rGQDs on the substrate electrode.

The measured impedance consists of electrode‐skin contact impedance and body impedance associated with the liver.^[^ ^6a‐c^ ^]^ This study aimed to reduce electrode‐skin contact impedance and acquire more accurate impedance signals associated with the liver to differentiate early‐stage NAFLD and healthy controls. Thus, we synthesized PtNPs@rGQDs onto the substrate electrode to reduce electrode‐skin contact impedance.^[^ ^26^ ^]^ The synthesis of PtNPs@rGQDs involved a two‐step electrochemical process where GQDs were first reduced onto substrate electrodes, and then PtNPs were electrochemically deposited, as illustrated in **Figure** **3**a. As seen in Figure 3b‐i, the PtNPs, with a size of ≈ 30 nm, are supported by well‐dispersed rGQDs. The EDX elemental mapping images indicated the presence of C and Pt elements (Figure 3b‐iv,v). The corresponding energy spectrum data are exhibited in Figure 3b‐vi. The peaks at 0.277 keV and 2.331, 9.442, 11.251, and 12.942 keV confirmed the presence of C and Pt, respectively (Figure 3b‐vi; Figure S5, Supporting Information).^[^ ^27^ ^]^ The TEM grid was made from copper, and a Cu element peak at 8.048 keV was observed. The results of the EDX elemental mapping images are consistent with the corresponding energy spectrum data, demonstrating that we succeeded in the synthesis of PtNPs@rGQDs on the sensing electrode surface using two‐step electrochemical depositions. From the high‐resolution TEM image of PtNPs@rGQDs (Figure 3b‐ii), the detected lattice distance of 0.23 nm corresponded well with the Pt (111) plane, known for its stable structures, thus contributing to the accurate collection of impedance signals for NAFLD detection.^[^ ^28^ ^]^ The electrochemical reduction of GQDs effectively adjusted the ratio of sp^2^‐hybridized carbon atoms and oxygen‐containing functional groups.^[^ ^12^, ^29^ ^]^ The oxygen‐containing functional groups and well‐dispersed structures provided additional anchoring sites for Pt NPs during subsequent electrochemical deposition. Raman measurements identified the sp^2^ domain (G‐band) and sp^3^ domain (D‐band). A higher ratio (1.09‐fold increase) of I_G_ to I_D_ indicated that more sp^2^ carbon atoms were generated after electrochemical reduction (Figure 3c), which is beneficial for faster charge transfer.^[^ ^30^ ^]^

We utilized X‐ray diffraction (XRD) measurement to analyze the crystal structures of the synthesized PtNPs@rGQDs (Figure 3d). The XRD peaks of PtNPs@rGQDs at 2*θ* of 39.8°, 46.2°, 67.5°, and 81.3° were indexed to the (111), (200), (220), and (311) planes of Pt, confirming the reliable deposition of PtNPs.^[^ ^31^ ^]^ We employed an X‐ray photoelectron spectroscopy (XPS) test to determine the valences of PtNPs@rGQDs. The C 1s spectrum showed four peaks at 288.4, 285.7, 285.0, and 284.5 eV, corresponding to C═O, C−N, C−C, and C═C bonds, respectively (Figure 3e). For the Pt 4f spectrum, the pairs of peaks at 74.3/71.0 and 74.9/71.7 eV were assigned to Pt^0^ and Pt^2+^, respectively (Figure 3f).^[^ ^32^ ^]^ These results demonstrated that the well‐dispersed rGQDs, with highly conductive sp^2^‐hybridized carbon and sufficient oxygen‐containing functional groups, can anchor Pt NPs tightly on the sensor to reduce electrode‐skin contact impedance.

We employed electrochemical impedance spectroscopy to analyze the deposited PtNPs@rGQDs (Figure 3g,h).^[^ ^33^ ^]^ It was observed that the intrinsic high impedance of the substrate electrodes, stemming from their limited active surface area, presented a significant barrier to accurate recordings (Figure 3g). The phase angle, reflecting the voltage‐current phase displacement, trends toward neutrality at higher frequencies, suggesting a predominance of capacitive behavior at the interface (Figure 3h).^[^ ^34^ ^]^ The electrochemical reduction of PtNPs@rGQDs onto the substrate electrode exhibited a significant impedance reduction due to the elevated specific capacitance of the composite layer. Beyond the 1 kHz threshold, the PtNPs@rGQDs phase angle plot approached nullity, signifying a capacitance‐dominated response conducive to electrical recordings (Figure 3h). The comparisons of impedance and phase angle between the unmodified substrate electrode, PtNPs‐modified electrode, and PtNPs@rGQDs‐modified electrodes across a spectrum of frequencies were detailed in Tables S1 and S2 (Supporting Information). Employing a multi‐frequency electrical impedance technique enables the detection of various tissues, given the distinct impedance properties of diverse tissue architectures.

Electrode‐skin contact impedance is the resistance encountered when electrical current flows from an electrode through the skin, significantly influenced by various factors such as the properties of the electrode material and the methods used to secure the electrode to the skin, including the application of adhesives to enhance contact.^[^ ^35^ ^]^ To minimize measurement impedance, it is important to ensure the skin‐electrode interfaces have high capacitance and are highly conductive. Strong adhesion to the skin is crucial for reducing measurement impedance. The reduction of electrode‐skin contact impedance is crucial for enhancing the quality of bioelectrical signal capture, as lower impedance leads to reduced noise and more precise readings. The utilization of as‐synthesized PtNPs@rGQDs nanomaterials has been shown to increase the contact area between electrodes and skin due to the unique nanostructures of PtNPs@rGQDs (Figure 3b). Concurrently, the adhesive layer ensures a robust bond between the electrodes and skin, enhancing contact by expanding the contact area. Accordingly, the double‐layer capacitance (*C_dl_*) was increased (Figure 3a, Randles equivalent circuit), thereby diminishing the electrode‐skin contact impedance (Formula 1).^[^ ^35^ ^]^ This optimized interaction between the electrode and skin not only improves signal fidelity but also paves the way for advancements in bioelectrical signal monitoring technologies. In this work, we demonstrated that the unique nanostructures of as‐synthesized PtNPs@rGQDs (Figure 3b) combined with adhesive layer design (Figure 2h‐i) are effective in reducing electrode‐skin contact impedance (Figure 3g,h). The electrode‐skin impedance can be derived as:^[^ ^35b^ ^]^

(1) $$Z(w)\;=\;R_{d}\;+\;\frac{1}{jwC_{dl}+\frac{1}{R_{e}}},\;w\;=\;2\pi f$$

### 2.4 An Attention‐Based Deep Learning Model to Classify Early‐Stage NAFLD and Healthy Controls

We used a mouse model to develop the disease model of early‐stage NAFLD via low‐density lipoprotein receptor knockout (*Ldlr^−/−^*) mice and a high‐fat diet regimen. Accordingly, we designed the on‐skin impedance sensor patch (≈42 mm × 28 mm × 0.2 mm) based on the mouse body size. The measured impedance includes electrode‐skin contact impedance and body impedance. We synthesized the PtNPs@rGQDs nanomaterial on sensing electrodes to reduce electrode‐skin contact impedance for highly accurate body impedance recordings. As shown in Figure S4 (Supporting Information), the measured impedance decreased (≈71.6% decline at 50 kHz) rapidly with the increase in electrode diameter from 1 to 3 mm. Although the measured impedance continued to decrease with the increase in electrode diameter from 1 to 4 mm, the decrease in measured impedance became smaller and may bring negative effects due to narrower electrode spacing, impairing the measurement of impedance in multi‐electrode sensors.^[^ ^36^ ^]^ Thus, we adopted the electrode with a diameter of 3 mm for impedance measurements.

**Figure 4:**
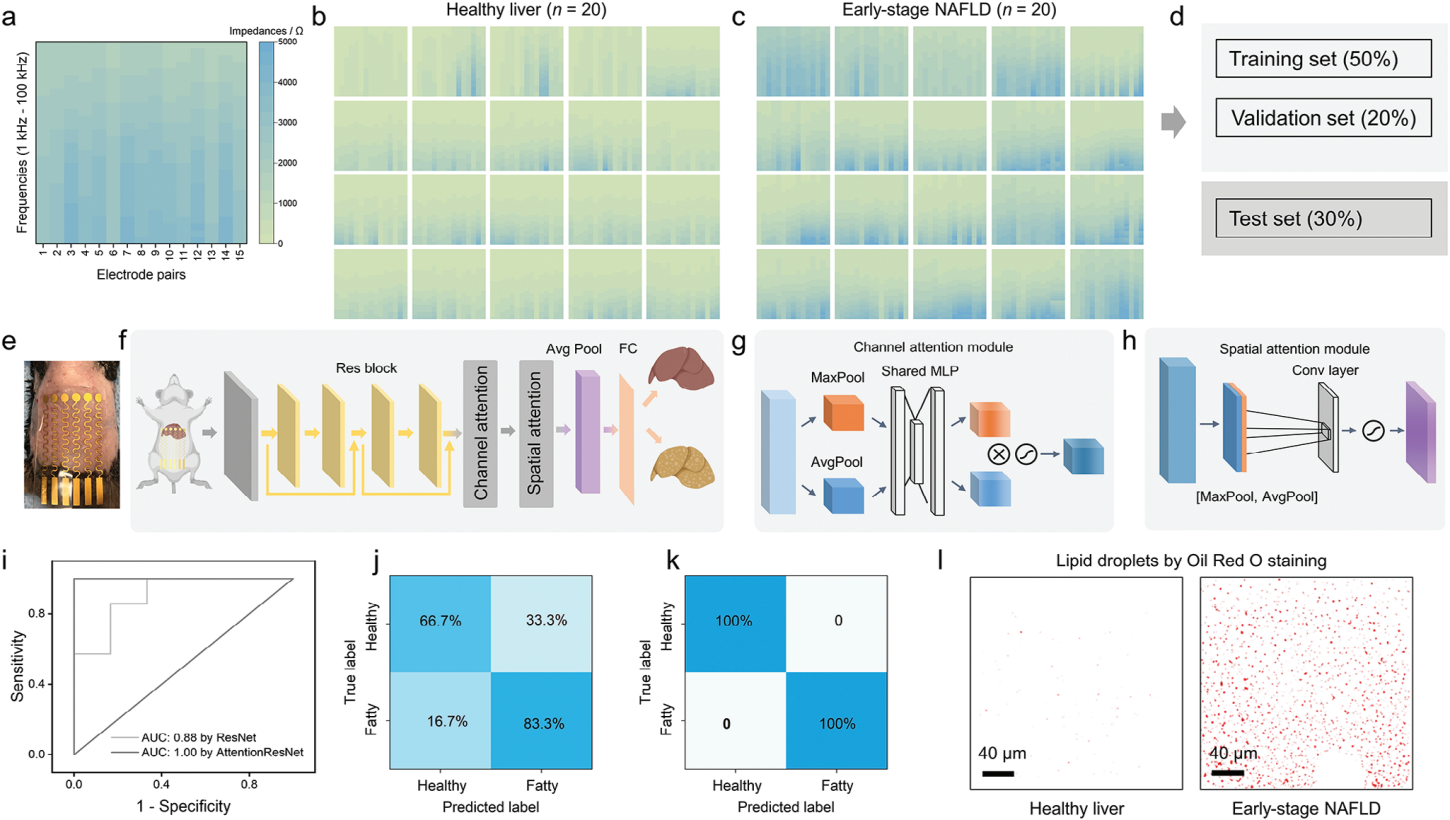
An attention‐based deep learning model to classify early‐stage NAFLD and healthy controls using impedance matrices obtained from an on‐skin sensor. a) A representative impedance matrix associated with the liver obtained from an on‐skin sensor near the liver region. b) Impedance matrices associated with the livers following a standard diet regimen (mouse number: n = 20). c) Impedance matrices from the livers subjected to a high‐fat diet regimen (mouse number: n = 20). d) Dataset partitioning ratio for training, validation, and testing sets. e) Actual detection photographs during animal experiments (electrode diameter: 3 mm). f) The architecture of the attention‐based deep learning model, including the on‐skin impedance sensor mounting location near the mouse liver. g,h) Illustrations of the channel g) and spatial h) attention mechanisms within the model. i) ROC curves comparing the performance of ResNet and AttentionResNet in detecting early‐stage NAFLD. j,k) Confusion matrices for early‐stage NAFLD detection using ResNet j) and AttentionResNet k). l) Validation of healthy and early‐stage NAFLD differentiation based on lipid droplet distribution, as revealed by Oil Red O histological staining.

In **Figure** **4**a, the impedance matrix associated with the liver was obtained using a fabricated on‐skin sensor over a multi‐frequency range of 1 to 100 kHz.^[^ ^9^, ^34^, ^37^ ^]^ Figure 4b,c illustrate the datasets from *Ldlr^−/−^* mice subjected to standard and high‐fat diets, respectively. To improve the fidelity of the impedance data associated with the liver, we engineered an on‐skin soft sensor with minimal electrode‐skin contact impedance. Furthermore, we developed an attention‐based deep learning model to differentiate between impedance signals related to early‐stage NAFLD and healthy controls, as shown in Figure 4e–g. The collected data encompassed forty 15 × 30 impedance matrices, corresponding to 20 early‐stage NAFLD cases (Figure 4c) and 20 healthy controls (Figure 4b). As depicted in Figure 4d, the datasets were divided into a training set (50%), a validation set (20%), and a test set (30%). To classify early‐stage NAFLD and healthy controls, we integrated a channel attention module (Figure 4f) and a spatial attention module (Figure 4g) into the Residual Neural Network (ResNet) architecture (Figure 4e, Figures S2 and S3, Supporting Information).^[^ ^15^, ^16^, ^38^ ^]^ Residual building blocks were implemented to mitigate the vanishing and exploding gradient problem.^[^ ^16b^ ^]^ In addition, we employed 1 × 1 convolutional layers to enhance network training efficiency and inference speed with fewer parameters.^[^ ^39^ ^]^

**Figure 5:**
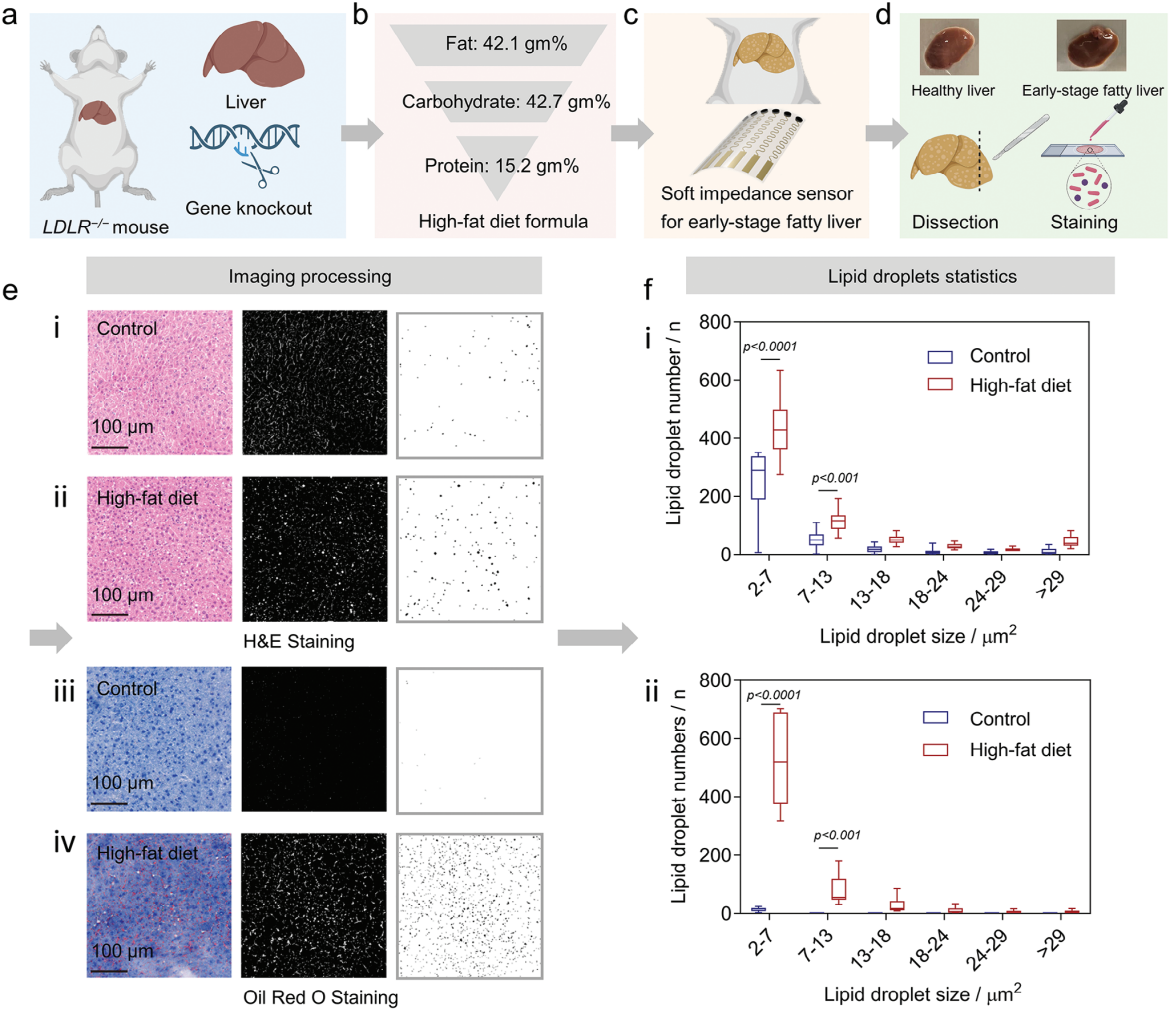
A comprehensive protocol was employed to validate the differentiation between healthy and early‐stage NAFLD. a) Genetic modification through *Ldlr^−/−^* gene knockout in mice. b) Implementation of a high‐fat diet regimen in *Ldlr^−/−^* mice for a duration of 4 weeks. c) Application of an on‐skin impedance sensor near the liver region. (d) Dissection and subsequent histological staining of liver tissues from both healthy and early‐stage NAFLD. e) Illustrative processed images from H&E staining (i: healthy liver, ii: early‐stage NAFLD) and Oil Red O staining (iii: healthy liver, iv: early‐stage NAFLD). f) Statistical analysis comparing the number of lipid droplets in early‐stage NAFLD from high‐fat diet‐induced *Ldlr^−/−^* mice to those in healthy controls (*p* < 0.0001 vs control for lipid droplet size from 2 to 7, *n* = 20; *p* < 0.001 vs control for lipid droplet size from 7 to 13, *n* = 20).

The confusion matrix for early‐stage NAFLD and healthy controls was calculated based on detection sensitivity and specificity, with ground truth labels derived from Oil Red O staining and lipid droplet distribution analysis (Figures 4k and 5).^[^ ^18b^ ^]^ The matrix confirmed that the AttentionResNet model correctly identified early‐stage NAFLD and healthy controls in the test set (Figure 4j). In contrast, the ResNet model yielded misclassification rates of 16.7% for early‐stage NAFLD and 33.3% for healthy controls (Figure 4i). The detection accuracy of the AttentionResNet model for early‐stage NAFLD reached above 97.5%, with an area under the receiver operating characteristic curve (AUC) of 1.0 (Figure 4h). The detection performances using the AttentionResNet model (AUC: 1.0) outperformed those using the ResNet model (AUC: 0.88), as shown in Figure 4h. These metrics underscore the effectiveness of the attention‐based deep learning algorithm in accurately categorizing high‐fat diet‐induced *Ldlr^−/−^* mice into early‐stage NAFLD with high accuracy.^[^ ^40^ ^]^ Thus, this attention‐based deep learning algorithm proved highly effective in detecting early‐stage NAFLD.^[^ ^7^, ^19^ ^]^

### 2.5 Validation of Early‐Stage NAFLD via Histological Staining and Statistical Analysis of Lipid Droplet Distribution

Here, we presented a comprehensive protocol for establishing a mouse model of early‐stage NAFLD using *Ldlr^−/−^* mice fed a high‐fat diet for four weeks (**Figure** **5**). We utilized an on‐skin impedance sensor for early NAFLD detection (Figure 5c). We validated it through hepatic dissection and histological staining (Figure 5d), along with a quantitative analysis of lipid droplet distribution (Figure 5f) after image processing (Figure 5e).^[^ ^18^, ^41^ ^]^

After the non‐invasive detection, the liver was sectioned and stained ex vivo using Hematoxylin & Eosin (H&E) and Oil Red O.^[^ ^42^ ^]^ H&E staining is a widely used histology method that allows for the differentiation of different types of tissues and cells. Oil Red O staining is a specialized technique that visualizes lipid droplets in cells and tissues.^[^ ^18b^ ^]^ The transformation of the H&E‐stained sections (Figure 5e‐i,e‐ii) and Oil Red O‐stained sections (Figure 5e‐iii,e‐iv) facilitated semi‐quantitative particle analysis. Fat‐free tissue is electrically conductive due to its high‐water and electrolyte content (including ions and proteins), whereas fatty tissue is less conductive due to its anhydrous properties.^[^ ^7^, ^19^ ^]^ The analyzed data demonstrated a significant increase in lipid droplets in the *Ldlr^−/−^* mice fed a high‐fat diet (Figure 5f). The observation was further supported by a substantial increase in the accumulation of small‐sized lipid droplets, suggesting early‐stage NAFLD. Electrical impedance variations resulting from the differing conductive properties of fatty and hydrated tissues were observed and illustrated in Figure 4b,c.

The alignment of lipid droplet quantifications with impedance recordings obtained from the fabricated on‐skin sensor validated the effectiveness of the proposed non‐invasive strategy for early‐stage NAFLD detection. Incorporating an attention‐based deep learning algorithm significantly enhanced the accuracy of NAFLD detection, thus facilitating early intervention. The proposed protocol, as seen in Figure 5, confirmed the early stage of NAFLD and demonstrated the effectiveness of on‐skin sensors in early detection.

## 3 Discussion

The non‐invasive detection of early‐stage NAFLD via electrical sensing represents a significant challenge. To overcome this challenge, we developed an adhesive and on‐skin impedance sensor that can conform to irregular body shapes and employed an attention‐based deep learning model. The deep learning model was designed to differentiate the impedance signals associated with early‐stage NAFLD and healthy controls.^[^ ^19^, ^38^, ^43^ ^]^ Our approach used the *Ldlr^−/−^* mouse model subjected to a high‐fat diet to establish a dataset of early‐stage NAFLD. We utilized histological staining to validate the progression of NAFLD. Our technique employed multi‐frequency impedance to measure deeper and organ‐specific impedances. This method reveals that organ‐derived signals have a stronger correlation with disease progression.^[^ ^19^ ^]^

A notable barrier to effectively measuring body impedance is the disturbance caused by electrode‐skin contact impedance. To mitigate this issue, we synthesized PtNPs@rGQDs to reduce electrode‐skin contact impedance.^[^ ^31^, ^44^ ^]^ This improvement is pivotal for advancing non‐invasive detection capabilities and could significantly enhance the detection and management of early‐stage NAFLD.

The primary recommendation for NAFLD therapeutics involves lifestyle modifications such as weight control, dietary supplements, exercise, which have proven to be the most effective approach.^[^ ^2^, ^45^ ^]^ Weight loss can decrease steatosis, although the sustained change was challenging to achieve.^[^ ^46^ ^]^ A diet low in carbohydrates and high in fiber and omega‐3 fatty acids could mitigate NAFLD in the long term, though the effects of short‐term dietary changes on the progression of the disease are yet to be determined.^[^ ^47^ ^]^ Exercise significantly enhances the management of NAFLD, with high‐intensity exercises yielding superior results, although such regimens are not advisable for individuals with underlying cardiovascular complications.^[^ ^46^ ^]^ Many enzymes are important in the processes of intracellular metabolism and lipotoxicity, notably fatty acid synthase (FAS) and acetyl‐CoA carboxylase (ACC). Peroxisome proliferator‐activated receptors (PPARs) facilitate lipid oxidation and the expression of fatty acid transport proteins (FATPs), positioning these molecules as potential therapeutic targets in the management of NAFLD. However, the drug treatment has different levels of side effects.^[^ ^2b^ ^]^ In addition to lifestyle adjustments and drug interventions, surgical options present a viable treatment pathway for critical NAFLD cases, particularly those facing obesity‐associated comorbidities such as cardiovascular diseases.^[^ ^48^ ^]^ Nonetheless, the inherent complications and risks associated with such invasive procedures restrict their applications across the broader patient population.

The development of deep learning, multiomics, and gut microbiota has improved the diagnosis, therapeutics, and management of NAFLD.^[^ ^49^ ^]^ The application of deep learning algorithms in the areas of precision diagnostics and prognostic assessment has attracted significant interest within the clinical field, warranting additional research in the context of recompensation for NAFLD‐related cirrhosis.^[^ ^49a^ ^]^ Machine learning algorithms often require substantial preprocessing and domain‐specific feature engineering to handle datasets. Human cognition dynamically focuses on pertinent information while disregarding irrelevant data.^[^ ^15b^ ^]^ Thus, we applied an attention mechanism that has shown promising results in various fields.^[^ ^22^, ^44^ ^]^ This study aimed to detect early‐stage NAFLD using an on‐skin impedance sensor. Impressively, our model achieved a prediction accuracy above 97.5%, underscoring the effectiveness of the attention mechanism for feature extraction in multi‐frequency impedance analysis. The integration of multiomics data has illuminated the genetic underpinnings of NAFLD. Investigating rare variants predicted to result in loss of function within genes of interest, alongside analyses of blood RNA expression and plasma proteomics, has identified potential causative genes and explored how alterations in their activity may play a role in disease pathogenesis.^[^ ^49b^ ^]^ Enhanced analytical techniques for distinguishing between healthy and unhealthy microbiomes, combined with a deep comprehension of the dietary and additional factors affecting the gut‐liver axis, will aid in the development of preventative measures and therapeutic approaches for this condition.^[^ ^49c^ ^]^

The present strategy is aimed at the binary classification of early‐stage NAFLD and healthy controls. The early detection of NAFLD is of paramount importance given its propensity to progress into non‐alcoholic steatohepatitis (NASH), liver cirrhosis, and liver cancer.^[^ ^50^ ^]^ Variations in liver compositions and cellular characteristics are pivotal in distinguishing between healthy and different liver diseases.^[^ ^50^ ^]^ The implementation of a multi‐electrode and multi‐frequency strategy combined with deep learning algorithms presents a feasible approach to accurately discern the progression of liver diseases. We have proposed a novel method that employs on‐skin impedance sensors coupled with attention‐based deep learning algorithms for distinguishing early‐stage NAFLD from healthy states. This approach holds the potential to facilitate the transition from binary to multi‐classification, thereby enabling the detection of various liver diseases at different stages. Despite the promising premise of the proposed method, it is important to note that experiments designed to validate the feasibility of a multi‐classification strategy for different liver diseases detection have yet to be conducted. Such validation is essential to ascertain the efficacy and reliability of the proposed method. Looking ahead, we envision significant expansions of the proposed detection method to encompass multi‐classification capabilities, thereby enhancing its utility in diagnosing a wider array of liver diseases. Moreover, exploring the integration of impedance sensors with portable circuits, Bluetooth technology, and battery power presents a viable pathway to eliminate reliance on bulky analytical devices. This evolution from on‐skin to wearable impedance sensors marks a critical step forward, enabling the continuous monitoring of physiological signals. Such advancements are particularly pertinent for the early detection of NAFLD in both asymptomatic and pre‐symptomatic individuals, offering a proactive approach to managing this increasingly prevalent liver condition.

Given the association of NAFLD with obesity, type 2 diabetes, and hypertension, the necessity for early detection and intervention is evident.^[^ ^51^ ^]^ Using an on‐skin electrical sensor combined with an attention‐based deep learning algorithm shows promise in improving the early detection of diseases. Our approach, which enables accurate and non‐invasive detection, offers the potential to improve patient prognoses and contribute to broader public health outcomes.

## 4 Experimental Section

### Materials

All chemical reagents were obtained from commercial sources. Toluene (≥99.5%), H_2_SO_4_ (≥97.0%), graphene quantum dots (1 mg mL^−1^), and chloroplatinic acid hydrate (H_2_PtCl_6_, ≥99.9% trace metals basis) were purchased from Sigma–Aldrich, USA. Styrene‐isoprene‐styrene (SIS, D1113) elastomer was acquired from Kraton Corporation. High‐tack silicone gel (Silbione RT Gel 4717 A/B) was purchased from Factor II, Incorporated. Ultrapure water (18.2 mΩ cm) was generated using a Millipore apparatus.

### Device Fabrication

First, an ≈ 200‐µm‐thick SIS substrate was cast from its solution in toluene onto a glass wafer modified with a PVA sacrificial layer (layer 1). Then, an Au connection was sputtered onto the SIS substrate using a pre‐processed mask via a laser cutting machine (ULTRA R5000, Universal Laser System) (layer 2). After that, the diluted silicone gel (Silbione RT Gel 4717 A/B) was sprayed onto the SIS substrate to encapsulate the Au connection, using a pre‐processed mask to shield the sensing electrodes (layer 3). Finally, the sensing electrode was coated with rGQDs and PtNPs through a two‐step electrochemical reduction process (layer 4).

### Characterizations

High‐resolution transmission electron microscopy images (HRTEM) and energy‐dispersive X‐ray (EDX) elemental mapping images were obtained on an FEI Titan TEM operated at 300 kV. X‐ray diffraction (XRD) analysis was performed on a Panalytical X'Pert Pro X‐ray Powder Diffractometer. Raman spectra were acquired from a Raman spectrometer (HORIBA Scientific) using a 488 nm laser. X‐ray photoelectron spectroscopy (XPS) measurements were conducted on a Kratos Analytical AXIS Ultra DLD photoelectron spectrometer.

### Mouse Disease Model

Mice homozygous for the *Ldlr^tm1Her^* knockout mutation were obtained from Jackson Laboratories (Strain 0 02207) to generate *Ldlr^tm1Her/tm1Her^* (*Ldlr^−/−^*) mice. All mice were fed a high‐fat diet for four weeks. Mice were fed a high‐fat diet (Teklad TD.88137, Envigo, Indianapolis, IN) composed of 42.1% fat, 15.2% protein, and 42.7% carbohydrates based on caloric content for four weeks. The diet was supplied as soft pellets and replaced every 2–3 days to ensure freshness. Animal experiments were performed in compliance with the UCLA Institutional Animal Care and Use Committee (IACUC) under animal welfare assurance number A3196‐01. The Animal Research Committee (ARC) reviewed all animal procedures performed at UCLA. The mice colony was housed in the facilities maintained by the UCLA Department of Laboratory Animal Medicine (DALAM).

### Electrical Measurements

Electrical impedance measurements were performed on mice following the approved ethical guidelines. Before the measurements, the depilation of the mouse fur was conducted as a preoperative hair removal procedure. Subsequently, an on‐skin soft sensor was carefully mounted on the skin near the liver area. The sensing electrodes were controlled by a homemade module, ensuring precise operation. Electrical impedance was meticulously measured across a frequency range from 1 to 100 kHz, with a 5 mV amplitude at the open‐circuit potential, using an impedance analyzer (E4980AL, Keysight). The impedance magnitudes were acquired at 30 data points per frequency decade to achieve detailed analysis, allowing for a comprehensive evaluation of the electrical properties at the targeted location. The detection time is ≈ 15 min for each mouse, including inhalation anesthesia of the mouse (≈2 min), hair removal (≈3 min), and electrical impedance measurements (≈10 min). The six‐electrode impedance measurements include 15 combinations for the electrode pair. For each electrode pair, 30 different frequencies were employed from 1 to 100 kHz.

### Machine Learning Algorithms

The machine learning framework consisted of ResNet, attention layers, and a fully‐connected layer.^[^ ^16b^ ^]^ Fully convolutional layers were utilized to accelerate the training and prediction processes by reducing the number of machine parameters.^[^ ^39^ ^]^ The attention module, including spatial and channel attention, was employed to extract representative features in the multi‐frequency impedance dataset.^[^ ^38^, ^43^ ^]^ The PyTorch torch‐metrics library was adopted to evaluate the performance of the proposed deep learning algorithm, with metrics such as ROC, Accuracy, and Confusion Matrix.^[^ ^52^ ^]^ The code for attention‐based machine learning is available in the GitHub repository (https://github.com/cardioAI/AttentionFattyLiver).

### Statistical Analysis

All data are presented as the mean ± standard error of the mean (SEM). The analysis included impedance results from 20 mice on a standard diet regimen and 20 mice on a high‐fat diet regimen. Statistical evaluation of the groups was conducted using a one‐way analysis of variance (ANOVA) to assess differences among groups, and a two‐way ANOVA to examine the effects under varying conditions. A *p*‐value of < 0.05 was considered statistically significant. All statistical analyses were carried out utilizing GraphPad Prism version 9.0.1 (GraphPad Software, Inc.).

## Conflict of Interest

The authors declare no conflict of interest.

## Author Contributions

The author's contribution is as follows: conceptualization (K.W., J.M.C., S.W., E.Z., T.K.H.); methodology (K.W., S.W., J.M.C., W.H., E.Z., Y.Z.); investigation (K.W., S.R., T.K.H.); visualization (K.W., J.M.C., S.W., J.Y.); supervision (T.K.H.); writing (K.W., T.K.H.), review (K.W., S.M., T.K.H.), and editing (K.W., B.A., P.Z., A.J., T.K.H.).

## Supporting information

Supporting Information

Supplemental Video 1

Supplemental Video 2

## Data Availability

The data that support the findings of this study are available on request from the corresponding author. The data are not publicly available due to privacy or ethical restrictions.
