# Supplementary material for: Non‐Invasive Detection of Early‐Stage Fatty Liver Disease via an On‐Skin Impedance Sensor and Attention‐Based Deep Learning
Source: Adv Sci (Weinh). 2024 Jun 17;11(31):2400596. doi: 10.1002/advs.202400596 (PMC11336938; doi:10.1002/advs.202400596)
Supplement: Supplementary file 1 — Supporting Information [file ADVS-11-2400596-s001.pdf]

ADVANCED SCIENCE
Open Access

Supporting Information

for *Adv. Sci.*, DOI 10.1002/advs.202400596

Non-Invasive Detection of Early-Stage Fatty Liver Disease via an On-Skin Impedance Sensor and Attention-Based Deep Learning

*Kaidong Wang, Samuel Margolis, Jae Min Cho, Shaolei Wang, Brian Arianpour, Alejandro Jabalera, Junyi Yin, Wen Hong, Yaran Zhang, Peng Zhao, Enbo Zhu, Srinivasa Reddy and Tzung K. Hsiai**

Supporting Information

Non-Invasive Detection of Early-Stage Fatty Liver Disease via an On-Skin Impedance Sensor and Attention-Based Deep Learning

*Kaidong Wang, Samuel Margolis, Jae Min Cho, Shaolei Wang, Brian Arianpour, Alejandro Jabalera, Junyi Yin, Wen Hong, Yaran Zhang, Peng Zhao, Enbo Zhu, Srinivasa Reddy, and Tzung K. Hsiai**

K. Wang, S. Margolis, J. Cho, P. Zhao, E. Zhu, T. K. Hsiai
Department of Medicine, David Geffen School of Medicine, University of California Los Angeles, Los Angeles, California, USA
E-mail: Thsiai@mednet.ucla.edu
K. Wang, S. Wang, B. Arianpour, A. Jabalera, J. Yin, Y. Zhang, T. K. Hsiai
Department of Bioengineering, Henry Samueli School of Engineering and Applied Sciences, University of California Los Angeles, Los Angeles, California, USA
W. Hong, E. Zhu
Department of Materials Science and Engineering, University of California Los Angeles, Los Angeles, California, USA
S. Reddy
Department of Molecular and Medical Pharmacology, University of California Los Angeles, Los Angeles, California, USA
K. Wang, T. K. Hsiai
Department of Medicine, Greater Los Angeles Veterans Affairs (VA) Healthcare System, Los Angeles, California, USA

Table of Contents

Figure S1. Equivalent circuit of measured impedance consisting of electrode-skin contact impedance and body impedance.
Figure S2. Training and validation loss curves of ResNet and AttentionResNet model to classify early-stage NAFLD and healthy controls.
Figure S3. Training and validation accuracy of ResNet and AttentionResNet model to classify early-stage NAFLD and healthy controls.

Figure S4. Comparison of measured impedance values on skin using electrodes of different diameters: 1, 2, 3, and 4 mm, with photographs displayed.

Figure S5. Energy spectrum in transmission electron microscopy (TEM) confirming the existence of C and Pt elements. TEM grid was made from copper, a Cu element peak was observed.

Table S1. Impedance values in different frequencies.

Table S2. Phase angles in different frequencies.

Video S1. Confocal image of early-stage fatty liver using Oil Red O staining.

Video S2. Confocal image of healthy liver using Oil Red O staining.

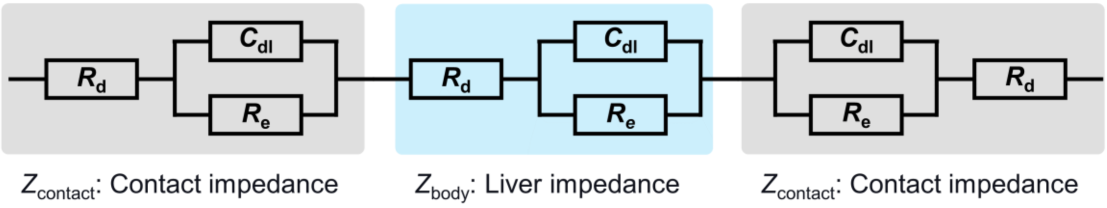

Figure S1. Equivalent circuit of measured impedance consisting of electrode-skin contact impedance and body impedance (Z: Impedance; C_{dl}: Double-layer capacitance; R_{ct}: Charge-transfer resistance).

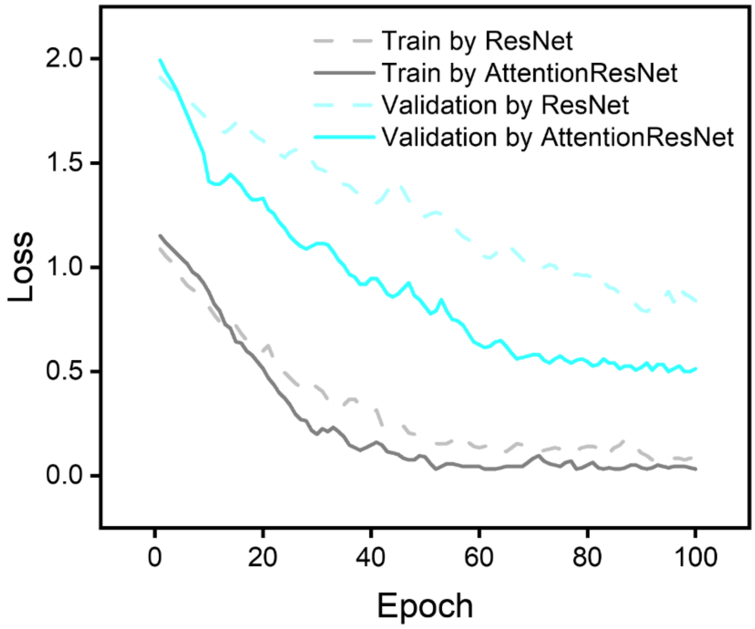

Figure S2. Training and validation loss curves of ResNet and AttentionResNet model to classify early-stage NAFLD and healthy controls.

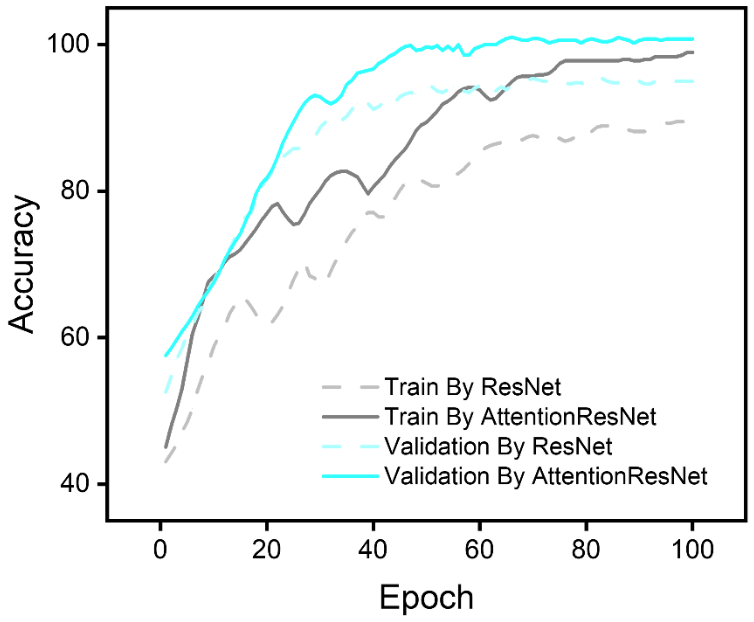

Figure S3. Training and validation accuracy of ResNet and AttentionResNet model to classify early-stage NAFLD and healthy controls.

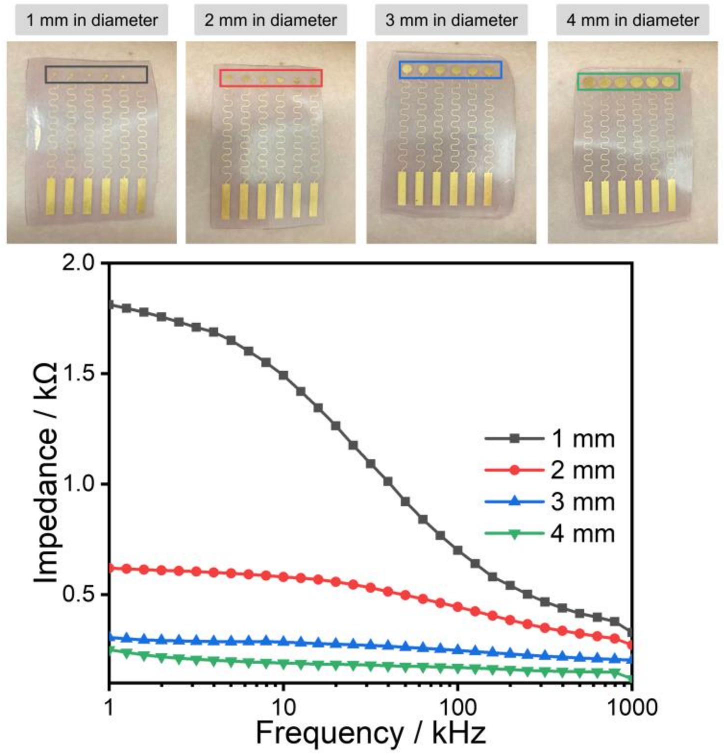

Figure S4. Comparison of measured impedance values on skin using electrodes of different diameters: 1, 2, 3, and 4 mm, with photographs displayed.

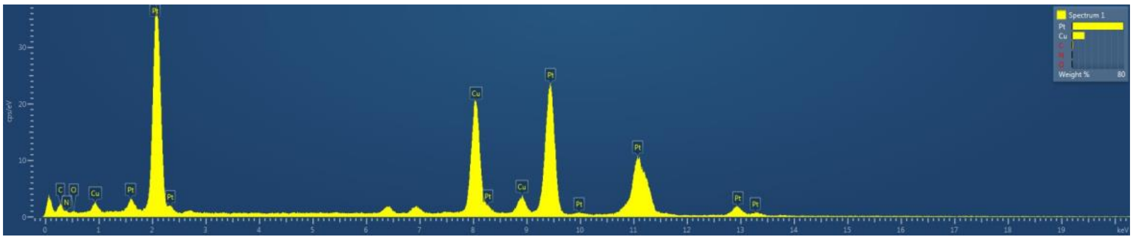

Figure S5. Energy spectrum in transmission electron microscopy (TEM) confirming the existence of C and Pt elements. TEM grid was made from copper, a Cu element peak was observed.

Table S1. Impedance values in different frequencies.

Frequency / kHz	1	5	10	25	50	100
Substrate / kΩ	3.355	2.720	2.109	1.307	0.899	0.640
PtNPs / kΩ	0.799	0.722	0.667	0.580	0.510	0.445
PtNPs@rGQDs/ kΩ	0.306	0.287	0.285	0.272	0.261	0.248

Table S2. Phase angles in different frequencies.

Frequency / kHz	1	5	10	25	50	100
Substrate / kΩ	-9.541	-25.155	-34.733	-41.459	-40.062	-35.448
PtNPs / kΩ	-10.822	-9.800	-11.825	-14.413	-15.480	-15.919
PtNPs@rGQDs/ kΩ	-17.228	-6.874	-5.827	-6.232	-7.010	-7.241
